# Age‐adapted eGFR thresholds underestimate the risks beyond kidney failure associated with CKD in older populations

**DOI:** 10.1111/jgs.18046

**Published:** 2022-09-24

**Authors:** Jennifer S. Lees, Michael G. Shlipak

**Affiliations:** ^1^ Institute of Cardiovascular and Medical Sciences University of Glasgow Glasgow UK; ^2^ Kidney Health Research Collaborative, Department of Medicine University of California San Francisco San Francisco California USA

## INTRODUCTION

Current guidelines define a diagnosis of chronic kidney disease (CKD) when glomerular filtration rate (GFR) falls persistently below a fixed threshold of 60 ml/min/1.73 m^2^, regardless of age.^1^ This approach has been criticized as it does not account for physiological aging, nor the lifetime risk of end‐stage kidney disease, which becomes less likely with increasing age due to the rising risks of death from other causes. Alternative, age‐adapted CKD thresholds were formally proposed by a group of experts in nephrology and GFR measurement in 2019,^2^ suggesting that the GFR threshold for diagnosis be increased to 75 ml/min/1.73 m^2^ in persons younger than 40 years, retained at 60 ml/min/1.73 m^2^ for those aged 40–64 years and lowered to 45 ml/min/1.73 m^2^ for those aged 65 years and older.

### Potential advantages of an age‐adapted CKD definition in older people

Proponents of an age‐adapted definition of CKD suggest two main arguments to support the adoption of lowered diagnostic thresholds in older people.^2^

1. Kidney senescence is commonly observed with increasing age. In healthy living kidney donors, increasing age over 35–40 years was associated with progressively lower nephron number and measured GFR (mGFR),^3^, ^4^ whereas single‐nephron GFR remained relatively constant.^3^ With increasing age, a degree of eGFR decline is expected: across a fixed eGFR threshold, a greater proportion of older adults will be diagnosed with CKD, even in otherwise healthy individuals.
2. Mortality risk is higher on average in older adults with GFR <45 ml/min, but not with GFR 45–60 ml/min, compared to those with GFR >60 ml/min. In a study reported in JAMA Internal Medicine, Liu et al.^5^ reported the estimated prevalence of CKD, and the subsequent incidence of kidney failure and death according to standard versus age‐adapted thresholds; and risks were compared with a reference group of patients considered not to have CKD (eGFR 60–89 ml/min/1.73 m^2^). In this study, changing thresholds for CKD diagnosis affected older adults in the following ways: age‐adapted thresholds identified a lower prevalence of CKD in the over 65 age group compared to the standard definition (decreased from 72.6% to 51.4%). The median age and eGFR at diagnosis went down (age decreased from 73 to 68 years and eGFR decreased from 53 to 43 ml/min/1.73 m^2^). Using the standard definition, individuals with an eGFR of 45–59 ml/min/1.73 m^2^ would be classified as having early‐stage CKD, however, they were found to have similar risks of kidney failure and death as those without CKD. In contrast, those with early CKD using the age‐adjusted definition had a higher risk of death and kidney failure compared to those without CKD. These findings appear reassuring that using a lower eGFR threshold that reduced the frequency of diagnosis of CKD in older adults would be unlikely to miss people at high risk of progression to kidney failure and may reduce medicalization of CKD; however, we are not aware of data that support improvements in quality of life or other patient‐centered outcomes by avoiding “overdiagnosis” of CKD in older people.

### Potential disadvantages of an age‐adapted CKD definition in older people

In our opinion, there are two major disadvantages to adopting age‐adapted thresholds that disproportionately affect older adults. First, that GFR is usually estimated using serum creatinine alone (eGFRcr), which performs progressively worse with higher age and frailty. Second, that the primary, preventable risk associated with CKD and CKD progression in older people is cardiovascular disease: particularly in the early stages of CKD, eGFRcr decline does not necessarily correlate with cardiovascular disease risk; other glomerular filtration markers are more sensitive and specific for detecting older adults at higher risk of cardiovascular disease. We will discuss each of these issues in turn, before considering our recommendations for the diagnosis and management of CKD in older patients.

### Serum creatinine alone overestimates GFR and underestimates risk in older adults

The most important determinants of serum creatinine at normal GFR are muscle mass and physical activity.^6^ Muscle mass and function both decline at older age, even in healthy individuals.^7^ eGFRcr equations includes an adjustment for age and sex, but incompletely account for differences in “average” loss of muscle mass with age: the populations in which the most popular equations were developed primarily included male participants in middle age.^8^, ^9^ Due to lower muscle mass and function with increasing age, serum creatinine therefore tends to overestimate GFR (and underestimate CKD)^6^ in older people.

Given the challenges of using serum creatinine alone in equations to estimate kidney function in older adults, international guidelines suggest additional, confirmatory testing for CKD using cystatin C.^10^ Cystatin C is the most widely studied alternative to serum creatinine for estimating kidney function, but is not currently widely used, primarily due to differences in cost and availability. Cystatin C is more expensive than serum creatinine: £2.50 (US $3) compared to £0.25 (US $0.30).^11^ However, with calls for wider implementation of cystatin C testing,^12^ costs may fall due to economies of scale and it may become more practical for smaller laboratories to offer this test routinely. Cystatin C has also been criticized as a marker of kidney function: serum levels may also be influenced by inflammation, obesity, diabetes, smoking and thyroid disease,^13^, ^14^, ^15^, ^16^ and eGFRcys may also capture aspects of cardiometabolic risk not directly related to kidney function. However, eGFR based on cystatin C offers a more accurate estimate of GFR than serum creatinine around the threshold for CKD diagnosis (60 ml/min/1.73 m^2^).^9^, ^17^, ^18^, ^19^ In older populations specifically, both eGFRcr‐cys and eGFRcys have been shown to be superior to eGFRcr for GFR estimation, and they improve classification of CKD across a threshold of 60 ml/min/1.73 m^2^.^17^, ^18^

### Kidney failure is not the primary risk associated with CKD

Cardiovascular disease and death are more common than kidney failure at all ages and all stages of CKD.^20^, ^21^ It is in the early stages of CKD where primary preventative treatments for cardiorenal complications, such as sodium‐glucose co‐transporter 2 inhibitors (SGLT2i), statins and anti‐hypertensives, are most likely to be effective. Early identification of CKD and recognition of the broader implications of this diagnosis is essential to optimize cardiorenal risk reduction strategies and protect older adults from preventable disease.

Around the threshold for CKD diagnosis (both above and below eGFRcr 60 ml/min/1.73 m^2^), eGFRcys offers enhanced risk stratification of kidney failure, death, and cardiovascular disease compared with eGFRcr,^22^ particularly stroke in older adults.^23^ Older adults with eGFRcys (but not necessarily eGFRcr) 45–59 ml/min/1.73 m^2^ have elevated risks for all of the important complications of CKD, particularly cardiovascular disease. Compared with eGFRcr levels of 45 ml/min/1.73 m^2^, similar values of eGFRcys are associated with nearly twice the age‐adjusted risk of cardiovascular disease and death.^21^, ^24^ eGFRcr‐cys also offers enhanced risk stratification compared with eGFRcr alone^21^, ^24^; however, the inclusion of serum creatinine in this equation appears to attenuate the ability to detect risk compared with eGFRcys.

### Recommendations

Although there is some rationale for tailoring the eGFR thresholds for CKD by age, these are restricted by the limitations of the creatinine test. We favor a modified approach.

**FIGURE 1:**
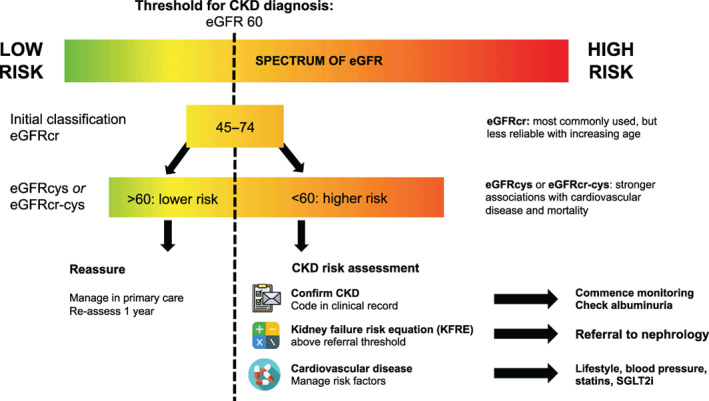
Schematic of the potential utility of cystatin C testing (as eGFRcys or eGFRcr‐cys) to risk‐stratify older adults with eGFRcr around the threshold of CKD diagnosis. SGLT2i: sodium‐glucose co‐transporter‐2 inhibitors

1. **Retain** **current** **thresholds** **for** **CKD** **diagnosis**.
  Simplicity is important. As for other chronic disease processes with risk‐based thresholds for diagnosis (such as hypertension), most CKD is managed in primary care. eGFR tends to decline with increasing age and so CKD is expected to become more common at older age. Retaining the existing, higher thresholds for CKD diagnosis in older people allows for an increased frequency and earlier diagnosis of CKD diagnosis at older age. An increased frequency of diagnosis increases awareness of CKD and encourages vigilance in monitoring (for example, improving albuminuria testing), but allows for some flexibility in approach. Drawing parallels with hypertension, management is undoubtedly different (and often less aggressive) in older people, acknowledging that the relative benefit of treatment may be lower, and there may be a higher risk of adverse effects associated with intensive treatment of hypertension. The same is true for CKD.
2. **Perform** **confirmatory** **testing** **for** **CKD** **using** **cystatin** **C** **in** **older** **adults**.
  In older adults (age >65 years) with eGFRcr 45–74 ml/min/1.73 m^2^, we recommend performing confirmatory testing for CKD using measures based on cystatin C (eGFRcys or eGFRcr‐cys). This is in keeping with (uncommonly implemented) recommendations from Kidney Disease Improving Global Outcomes (KDIGO) international guidelines for the diagnosis and management of CKD.^10^ Sending a single serum value of cystatin C allows the flexibility to incorporate this filtration marker into various equations.
  Confirmatory testing using eGFRcys or eGFRcr‐cys, around the threshold of 60 ml/min/1.73 m^2^, could yield two main advantages relative to changing the thresholds for diagnosis. First, equations incorporating cystatin C improves the specificity of CKD diagnosis and the accuracy of GFR estimation in older individuals,^18^ with implications for drug dosing and avoidance of nephrotoxins. Although cystatin C testing is expected to identify a proportion of older adults with CKD who were not identified using eGFRcr alone (therefore qualifying some additional older adults for further monitoring, treatments or referral to specialty care) cystatin C testing as eGFRcys or eGFRcr‐cys may also de‐classify a proportion of older adults from the CKD definition, achieving one of the primary aims of age‐adapted CKD thresholds.^2^ Further confirmatory work in diverse older populations would be welcomed. Second, compared to eGFRcr alone, cystatin C testing (eGFRcys or eGFRcr‐cys) improves detection of high‐risk CKD around the threshold for CKD diagnosis, identifying older individuals who are more likely to benefit from early intervention for cardiovascular disease, kidney failure, and premature death. Importantly, around the threshold for CKD diagnosis (GFR 45–74 ml/min/1.73 m^2^), cystatin C testing will also re‐classify a proportion of older adults with eGFRcys or eGFRcr‐cys >60 ml/min/1.73 m^2^ as lower risk, allowing direction of risk reduction strategies and specialty care resources to those at greatest risk for CKD complications (Figure 1).
  We would recommend eGFRcr‐cys to improve accuracy of GFR estimation and to guide drug dosing. We would recommend eGFRcys alone for risk assessment of cardiovascular disease, and mortality. Though detailed discussions of cost implications are beyond the scope of this commentary, the reduction in healthcare costs (including hospitalizations and procedures) associated with cardiovascular events may offset the additional cost of cystatin C testing and primary prevention strategies.
3. **Individualize** **risk** **assessment** **.**
  After understanding that individuals with eGFRcys or eGFRcr‐cys <60 ml/min/1.73 m^2^ are generally at higher risk of CKD complications, clinicians can further estimate individual risk. A variety of calculators have been developed and validated in large, multi‐center cohorts which determine individualized risk of incident CKD, kidney failure, cardiovascular disease and mortality (https://ckdpcrisk.org). In an era when precision medicine is increasingly pursued, the ability to quantify the risk associated with reductions in kidney function is more informative to patients, and to practitioners, than a label of present or absent CKD.
  The kidney failure risk equation (KFRE) quantifies 5‐year risk of kidney failure requiring renal replacement therapy in people with eGFR <60 ml/min/1.73 m^2^. This equation has been validated and recalibrated in an older cohort (average age 75 years) of people with CKD in primary care,^25^ identifies a greater proportion of older individuals who may benefit from nephrology specialty care referral than by using eGFR thresholds alone^26^ and may reduce nephrology wait times.^27^ In the UK, updated guidelines (2021) recommended referral to a nephrologist when 5‐year KFRE is >5%. Though other national and international groups have not yet recommended KFRE thresholds to guide nephrology referrals, it is likely that KFRE will feature more prominently soon (KDIGO guidelines are due to be updated in 2023).
  Among older adults who do not qualify for referral to nephrology specialty care, eGFRcys more sensitively identifies and more appropriately stratifies those who are at higher risk of cardiovascular disease^21^, ^24^ and who may benefit from interventions to reduce cardiovascular risk (Figure 1).

## CONCLUSION

“eGFRcr around the threshold for diagnosis of CKD is less accurate, particularly in older people. Older people with eGFRcr 45–59 ml/min/1.73 m^2^ are not necessarily at low risk of CKD complications.”

In the group of patients with eGFRcr 45–59 ml/min/1.73 m^2^, there are individuals who are both at higher and lower risk of cardiovascular disease, kidney failure and premature death. Cystatin C testing (eGFRcys or eGFRcr‐cys) can be used to: (i) improve specificity of diagnosis of CKD in older people; and (ii) stratify the risk of cardiovascular disease, kidney failure, and death in older patients. With longer life expectancy and an aging population, we should be adapting our testing strategies to suit the needs of our older patients, rather than changing patient care to adapt to the limitations of the creatinine test.

## AUTHOR CONTRIBUTIONS

Jennifer S. Lees wrote the first draft of the manuscript. Michael G. Shlipak critically advised on content and edited the manuscript. Both authors approved the final submitted version. Michael G. Shlipak acknowledges membership of the Kidney Disease Improvement Global Outcomes (KDIGO) guideline group for the evaluation and management of CKD.

## FUNDING INFORMATION

J.S.L. is funded by a Chief Scientist Office (Scotland) Postdoctoral Lectureship Award (PCL/20/10).

## CONFLICT OF INTEREST

The authors declare no conflicts of interest relevant to the submitted work. Outside the submitted work, J.S.L. has received personal honoraria from Pfizer, Bristol Myers Squibb, and Astra Zeneca.

## SPONSOR'S ROLE

J.S.L. acknowledges funding from a Chief Scientist Office (Scotland) Postdoctoral Lectureship Award (PCL/20/10). The funder had no role in the manuscript.

## References

[1] Kidney Disease Working Group . Kidney Disease: Improving Global Outcomes (KDIGO) 2012 clinical practice guideline for the evaluation and management of chronic kidney disease. Kidney Int. 2013;3:1‐150. 10.1038/ki.2013.243 23989362

[2] Delanaye P , Jager KJ , Bökenkamp A , et al. CKD: a call for an age‐adapted definition. J Am Soc Nephrol. 2019;30:1785‐1805. 31506289 10.1681/ASN.2019030238 PMC6779354

[3] Denic A , Mathew J , Lerman LO , et al. Single‐nephron glomerular filtration rate in healthy adults. N Engl J Med. 2017;376:2349‐2357. 28614683 10.1056/NEJMoa1614329 PMC5664219

[4] Fenton A , Montgomery E , Nightingale P , et al. Glomerular filtration rate: new age‐ and gender‐ specific reference ranges and thresholds for living kidney donation. BMC Nephrol. 2018;19:336. 30466393 10.1186/s12882-018-1126-8 PMC6249883

[5] Liu P , Quinn RR , Lam NN , et al. Accounting for age in the definition of chronic kidney disease. JAMA Intern Med. 2021;181:1359‐1366. 34459844 10.1001/jamainternmed.2021.4813 PMC8406213

[6] Nankivell BJ , Nankivell LFJ , Elder GJ , Gruenewald SM . How unmeasured muscle mass affects estimated GFR and diagnostic inaccuracy. EClinicalMedicine. 2020;29–30:100662. 10.1016/j.eclinm.2020.100662 PMC7788434 33437955

[7] Suetta C , Haddock B , Alcazar J , et al. The Copenhagen Sarcopenia Study: lean mass, strength, power, and physical function in a Danish cohort aged 20–93 years. J Cachexia Sarcopenia Muscle. 2019;10:1316‐1329. 31419087 10.1002/jcsm.12477 PMC6903448

[8] Levey AS , Stevens LA , Schmid CH , et al. A new equation to estimate glomerular filtration rate. Ann Intern Med. 2009;150:604‐612. 19414839 10.7326/0003-4819-150-9-200905050-00006 PMC2763564

[9] Inker LA , Schmid CH , Tighiouart H , et al. Estimating glomerular filtration rate from serum creatinine and cystatin C. N Engl J Med. 2012;367:20‐29. 22762315 10.1056/NEJMoa1114248 PMC4398023

[10] Kidney Disease: Improving Global Outcomes (KDIGO) Transplant Working Group . Chapter 1: definition and classification of CKD. Kidney Int Suppl. 2013;3:19‐62. 10.1038/kisup.2012.64 PMC4089693 25018975

[11] National Institute for Health and Care Excellence, NICE . Costing statement: chronic kidney disease implementing the NICE guideline on chronic kidney disease (CG182).

[12] Delgado C , Baweja M , Burrows NR , et al. Reassessing the inclusion of race in diagnosing kidney diseases: an interim report from the NKF‐ASN Task Force. Am J Kidney Dis. 2021;78:103‐115. 33845065 10.1053/j.ajkd.2021.03.008 PMC8238889

[13] Panaich S , Veeranna V , Zalawadiya S , Kottam A , Afonso L . Association of cystatin C with measures of obesity and its impact on cardiovascular events among healthy U.S. adults. J Am Coll Cardiol. 2013;61:E1420. 10.1089/met.2014.0018 25118891

[14] Goede DL , Wiesli P , Brändle M , et al. Effects of thyroxine replacement on serum creatinine and cystatin C in patients with primary and central hypothyroidism. Swiss Med Wkly. 2009;139:339‐344. 19529992 10.4414/smw.2009.12654

[15] Rule AD , Bailey KR , Lieske JC , Peyser PA , Turner ST . Estimating the glomerular filtration rate from serum creatinine is better than from cystatin C for evaluating risk factors associated with chronic kidney disease. Kidney Int. 2013;83:1169‐1176. 23423253 10.1038/ki.2013.7 PMC3661736

[16] Anderson A , Yang W , Hsu C‐Y , et al. Estimating GFR among participants in the Chronic Renal Insufficiency Cohort (CRIC) Study. Am J Kidney Dis. 2012;60:250‐261. 22658574 10.1053/j.ajkd.2012.04.012 PMC3565578

[17] Fan L , Levey AS , Gudnason V , et al. Comparing GFR estimating equations using cystatin C and creatinine in elderly individuals. J Am Soc Nephrol. 2015;26:1982‐1989. 25527647 10.1681/ASN.2014060607 PMC4520174

[18] Schaeffner ES , Ebert N , Delanaye P , et al. Two novel equations to estimate kidney function in persons aged 70 years or older. Ann Intern Med. 2012;157:471‐481. 23027318 10.7326/0003-4819-157-7-201210020-00003

[19] Inker L , Eneanya N , Coresh J , et al. New creatinine‐ and cystatin C–based equations to estimate GFR without race. N Engl J Med. 2021; 385:1737‐1749. 34554658 10.1056/NEJMoa2102953 PMC8822996

[20] Keith DS , Nichols GA , Gullion CM , Brown JB , Smith DH . Longitudinal follow‐up and outcomes among a population with chronic kidney disease in a large managed care organization. Arch Intern Med. 2004;164:659‐663. 15037495 10.1001/archinte.164.6.659

[21] Lees JS , Welsh CE , Celis‐Morales CA , et al. Glomerular filtration rate by differing measures, albuminuria and prediction of cardiovascular disease, mortality and end‐stage kidney disease. Nat Med. 2019;25:1753‐1760. 31700174 10.1038/s41591-019-0627-8 PMC6858876

[22] Peralta CA , Shlipak MG , Judd S , et al. Detection of chronic kidney disease with creatinine, cystatin c, and urine albumin‐to‐creatinine ratio and association with progression to end‐stage renal disease and mortality. JAMA. 2011;305:1545‐1552. 21482744 10.1001/jama.2011.468 PMC3697771

[23] Kühn A , van der Giet M , Kuhlmann MK , et al. Kidney function as risk factor and predictor of cardiovascular outcomes and mortality among older adults. Am J Kidney Dis. 2021;77:386‐396. 33197533 10.1053/j.ajkd.2020.09.015

[24] Shlipak MG , Matsushita K , Ärnlöv J , et al. Cystatin C versus creatinine in determining risk based on kidney function. N Engl J Med. 2013;369:932‐943. 24004120 10.1056/NEJMoa1214234 PMC3993094

[25] Major RW , Shepherd D , Medcalf JF , Xu G , Gray LJ , Brunskill NJ . The kidney failure risk equation for prediction of end stage renal disease in UK primary care: an external validation and clinical impact projection cohort study. PLoS Med. 2019;16:e1002955. 31693662 10.1371/journal.pmed.1002955 PMC6834237

[26] Bhachu HK , Cockwell P , Subramanian A , et al. Impact of using risk‐based stratification on referral of patients with chronic kidney disease from primary care to specialist care in the United Kingdom. Kidney Int Rep. 2021;6:2189‐2199. 34386668 10.1016/j.ekir.2021.05.031 PMC8343777

[27] Whitlock RH , Chartier M , Komenda P , et al. Validation of the kidney failure risk equation in Manitoba. Can J Kidney Health Dis. 2017;4:2054358117705372. 28491341 10.1177/2054358117705372 PMC5406122

